# Fighting the COVID-19 Crisis: Debt Monétisation and EU Recovery Bonds

**DOI:** 10.1007/s10272-020-0907-z

**Published:** 2020-07-28

**Authors:** Alberto Botta, Eugenio Caverzasi, Alberto Russo

**Affiliations:** 1 Dep. of International Business and Economics, Business Faculty, University of Greenwich - Faculty of Business, Old Royal Naval Coll., Park Row, SE10 9LS London, UK; 2 Department of Economics, Università degli Studi dell’Insubria, Via Ravasi 2, 21100 Varese, Italy; 3 Department of Economics, Universitat Jaume I, Av. Vicent Sos Baynat, s/n, 12071 Castellòn de la Plana, Spain

## Abstract

This paper highlights some peculiar characteristics of the economic crisis induced by the spread of COVID-19. It suggests two intertwined policy measures in order to tackle the emergency phase of the crisis and to support the economy in the subsequent recovery phase. The proposed short-term policy measures offer policy responses in the event of a second wave of coronavirus infections in the coming months. In the aftermath of the emergency phase, the current proposal puts forward the implementation of a massive EU-wide recovery plan addressing the long-lasting technological and environmental challenges of these years, which will be financed by European institutions through the issuance of European Pandemic Recovery Bonds.

The spread of COVID-19 has initiated major changes in governments' and central banks' policies. There is consensus among economists that the governments in Europe and in the US, i.e. the current epicentres of the pandemic, will have to take extraordinary measures in order to deal with the disruptive economic consequences of the coronavirus crisis. The overwhelming pressure on healthcare systems and the forced halt of economic activities require massive urgent emergency action to tame the immediate consequences of the crisis. Following the emergency phase, governments will need to implement further interventions in order to "prevent a recession morphing into a prolonged depression" (Draghi, 2020).

**Alberto Russo**, Universitat Jaume I, Castellòn de la Plana, Spain; and Università Politecnica delle Marche, Ancona, Italy.

Bold government interventions might imply a considerable increase in public debt. However, financial concerns should not limit governments' actions as the cost of hesitation may be dramatic both in terms of present and future social well-being. Concerns over the implications for the European public balance sheets may be justified, but they completely vanish in the face of the major damages that may be incurred by the European real economy. Sound public finance ultimately depends on a robust real economy.

In this paper, we first recommend a strong emergency response in which eurozone governments would cover operative costs of companies (small and medium firms in particular) and guarantee income flows to households in the context of a 'suspended' economy. In other words, "[t]he job is maintaining the economy on life support during a period of an artificially induced coma while we address the public health challenge" (Tooze, 2020). We then suggest the implementation of an EU-wide recovery plan based on public investment and addressing the not-to-be-forgotten climate crisis and the now well understood needs of our healthcare systems. We propose that these interventions be financed by two sets of bonds:

- bonds issued by the national governments to cover emergency costs, to be fully monetised and subsequently written off by the European Central Bank (ECB), in order to prevent any emergency-related increase in public debt stocks;

- recovery bonds to be issued by European institutions to relaunch the European economy in the immediate aftermath of the health crisis.

The first point is indispensable, urgent and might perhaps help to overcome the existing contrast among eurozone governments (even though it implies other types of institutional changes). The second is equally relevant, and indeed much needed, even regardless of the current crisis, though there is a little more time for discussion.

## Three crucial aspects of the COVID-19 economic shock

First, there is no doubt that the COVID-19 economic shock is a truly *exogenous* one. It does not depend on the will or previous misbehaviour of any government or private sector. This is a significant difference with respect to the frequently cited 2007-08 financial crisis. Indeed, the outbreak of this crisis was due to new practices in the financial sector, e.g. the emergence of so-called ‘shadow banking’, which was in turn closely connected to long-term developments in advanced economies, rising inequality first and foremost (Botta et al., 2019). European economies were initially affected by the worldwide financial meltdown because European banks were actively engaged in the diffusion of ‘toxic’ new financial products or supported unsustainable processes such as housing bubbles in Ireland, Spain and Greece. That crisis then morphed into the eurozone sovereign debt crisis due to (external) imbalances among eurozone countries, endogenously built-up in the initial phase of monetary integration (1999-2007), and the institutional deficiencies in monetary and, especially, fiscal policy (i.e. the pro-cyclicality of austerity measures during recessionary phases).

Second, the COVID-19 economic shock stands out as a *common* shock affecting all eurozone countries. While the timing and the size of the shock may slightly differ from country to country, there is little doubt that all of the eurozone economies will experience a recession.

Third, the economic crisis that Europe faces consists of a *complex mix of supply and demand shocks*. On the supply side, the restrictive measures taken by governments in order to implement social distancing and contain the spread of the virus have stopped or decreased production. Restrictions on mobility and firms’ functionality have simultaneously induced a tremendous drop in aggregate demand. If there is something that European policymakers should have learned from the global and the eurozone crises, it is that the recovery is much slower with no management of aggregate demand. The acute contraction in aggregate demand may well explain why inflation spikes due to supply constraints do not represent a serious threat at the moment; instead, even more worrisome deflationary trends appear more likely (De Grauwe, 2020).

## A brief look at some existing proposals

There is a growing debate among economists about the most appropriate monetary and fiscal measures for tackling such an extraordinary situation. Some of these have been already announced by national governments, the European Commission and the ECB.

A first proposal comes from previous ECB president Mario Draghi (2020), who emphasises the importance of financial institutions accommodating all credit requests from the private business sector in order to avoid firms’ bankruptcies and reductions in the employment level. In this sense, Draghi (and many others, e.g. Bénassy-Quéré et al. 2020) welcome the ECB’s recent decision to extend long-term refinancing operations, to expand quantitative easing (which may help large corporations to issue corporate bonds at a cheap rate), to reduce the main refinancing rate for banks to below zero (de facto subsidising their activity) and to temporarily slacken banks’ capital requirements. In their view, all these measures may help banks to expand lending as much as possible and at very low interest rates, possibly close to zero. Nevertheless, governments might have to intervene by compensating borrowers and de facto bailing out private companies by moving private liabilities to an expanding public balance sheet. Together, with extraordinary measures already taken in order to support healthcare systems, this will obviously imply that “much higher public debt levels will become a permanent feature of our economies and will be accompanied by private debt cancellation” (Draghi, 2020).

Economists all agree that governments should massively intervene via public expenditures; consequently, EU member states temporarily suspended the Stability and Growth Pact (SGP). Their views, however, diverge when it comes to the financing of much larger fiscal deficits of eurozone countries.

The ECB has already taken a fundamental step in the right direction by creating the Pandemic Emergency Purchase Programme (PEPP). This adds €780 billion to existing quantitative easing and makes the issuance of new public bonds easier and cheaper on financial markets (overcoming the 33% limit on a single country’s bond share, based on the capital key with some deviations). Given such a favourable monetary context, some economists think that the creation of long-term (50, 100 years or even perpetual) eurobonds issued by single member countries but jointly guaranteed by the tax capacity of the eurozone as a whole would be the best solution (Giavazzi and Tabellini, 2020; Bénassy-Quéré et al., 2020). Alternatively, the expanded asset purchasing programme recently announced by the ECB could represent the proper framework for the introduction of a 'European safe asset' possibly issued by a European institution (the European Commission, for example) rather than by national governments, and backed by a centralised taxation scheme, i.e. revenues from a newly created European tax or compulsory transfers from member states to the centre (Codogno and van den Noord, 2020). One reason for such centralised eurobonds,[1] among many others, should be the creation of fiscal space for stabilisation policies in times of major crises.

In a similar vein, the European Commission has announced the creation of SURE, a EU-level financial scheme supporting member states in the emergency provision of short-term work schemes such as *Cassa Integrazione Guadagni* in Italy and *Kurzarbeit* in Germany. Indeed, the main goal of SURE is to reduce EU member states single-country reliance on financial markets by partially replacing potentially costlier new issuances of national bonds with cheaper temporary loans provided by the European Commission, and in turn financed by the introduction of a common European AAA-rated asset.

The above proposals share a common aspect: They all foresee emergency plans grounded in the functioning of financial markets. While market mechanisms are suspended and cannot work for most of the real economy, its financial needs should still remain satisfied by the 'normal' provision of credit from financial institutions. This aspect is not trivial. In fact, it implies that at the end of the emergency phase, private companies and/or the public sector might face a higher stock of debt, albeit at reduced or no (interest) cost. And this may in turn weaken the effectiveness of recovery measures implemented in the post-pandemic period in fragile economies overburdened by newly created emergency-related debt.

While frequently treated separately, the emergency and post-pandemic phases of the current crisis are closely connected. Galì (2020) recognises this fact and suggests an alternative that relies on so-called helicopter money. This may take the form of either direct money transfers from central banks' accounts to citizens' bank accounts, generally referred to as 'direct cash handouts', or by 'monetary financing' of government expenditures by providing governments with grants. This has the advantage of central banks creating all the needed resources to deal with the emergency but not creating extra debt.

## An integrated policy package for the emergency and economic recovery

Our proposal addresses both the emergency and post-pandemic phases of the coronavirus crisis. The acute phase of the COVID-19 pandemic seems to be over in most European countries. Accordingly, they have lifted the most stringent lockdown measures implemented in March and April. The short-term policy measures described below can thus be interpreted as potential policy responses should a second wave of coronavirus infections occur in the coming months.

### Short-term actions to sustain the 'suspended' economy

In the event of a second wave of COVID-19 and a new lockdown, eurozone governments should step in to secure the incomes of a large part of the private sector. The idea is to accept that the normal functioning of eurozone economies might be de facto suspended again. While continuing to remunerate public servants, all businesses requiring support should receive government assistance covering around 70-80% of their labour costs (up to a predetermined ceiling) and the full amount of their fixed costs according to recent administrative/fiscal data. This should be done in favour of all businesses forced to close and of those that are still active but experiencing a major reduction in demand. Different types of support can be linked to different types of conditionalities, such as a no-layoff clause for employees benefitting from temporary employment protection schemes, or workers' involvement in the co-direction of companies benefitting from temporary equity from the public sector. Government expenditure should replace lacking demand during possible future emergency periods. Government transfers should also be directed towards self-employed and freelance workers unable to work in a new lockdown. Extra compensation should be remunerated to active workers employed in vital sectors, from hospitals to food, energy, communications, etc.

### Financing the emergency spending

Our proposal is in line with the idea of governments as the 'buyers of last resort' (see Saez and Zucman, 2020). Differently from Saez and Zucman (2020), however, we stress that the financing of these measures should come from the ECB and not from a tax increase, even not for the wealthiest. Even though more progressive taxation would

1 Codogno and van den Noord (2020) also propose that centralised eurobonds should replace national bonds in the balance sheets of financial institutions in their role as collaterals with seniority in refinancing operations with the ECB and in inter-bank transactions.

be highly desirable across Europe, the risk of a recessive effect must be avoided during emergency phases.

Specifically, we foresee a scheme according to which emergency spending by eurozone governments should be certified by the European Commission based on shared rules. Such certification from the EU Commission can effectively replace 'weak' conditionality associated with the emergency credit line from the ESM. Governments should then finance their crisis response by issuing public bonds that the ECB directly purchases on the primary market (see De Grauwe, 2020) and subsequently writes off from its own balance sheet. In doing so, the ECB will de facto make a transfer to the accounts of eurozone governments in order to provide them with the resources needed to tackle the current economic emergency (see Galì, 2020). This way, it will avert further increases in public debt stocks that could restrain governments' efforts to boost economic recovery.

The implementation of this financing scheme would certainly represent a violation of the ECB statute. If such an economic taboo cannot be challenged openly, it should be addressed implicitly. The ECB's purchases of government bonds on primary markets could take place indirectly via the creation of a public special purpose vehicle. This financial institution would buy bonds from governments on the primary market, and indirectly pass them to the ECB by issuing liabilities that the ECB itself can purchase in the context of ECB quantitative easing. Eventually, when public bonds issued during the crisis mature, they should be automatically rolled over (effectively becoming consols, i.e. perpetual, non-redeemable bonds) and, in any case, they should never be included in the computation of the debt-to-GDP ratios.

It is worth mentioning that such a proposal overcomes the political problem of the mutualisation of public debt. And it certainly implies no less challenging temporary amendments in the relationship between the ECB and national governments. Nonetheless, the close cooperation between governments and the ECB is vital for keeping the eurozone economy going and for avoiding financial speculations during possible future lockdown periods.

## Relaunching the economy in the aftermath of the emergency

When the situation allows for a gradual return to social life and for a restart of the private sector, European institutions should take a second step to support the recovery of the eurozone (one that was already needed even before the outbreak of the COVID-19 pandemic). A large-scale plan for financing physical and digital infrastructures, healthcare and scientific research, energy-saving and clean technologies along an ecological transition is needed. Emergency financing should not come at the expense of the other financial and developmental programmes of the EU, particularly the European Green Deal, the European Social Fund, the European Investment Fund, Horizon Europe and other cohesion funds. Rather, those programmes should be used to reignite the economy in a timely fashion.

In the case of the eurozone, the COVID-19 crisis is hitting a limping economic system, lagging behind in the evolution of key sectors (e.g. the automotive sector) and characterised by very low levels of public investment.[2] At the global level, the pandemic is taking place in the midst of an ecological crisis. The goal of a European recovery plan should thus not be limited to jumpstarting economic activity, but rather should guide the economy of the EU, and of the eurozone in particular, towards a more sustainable, technologically advanced and inclusive socio-economic system.

On the one hand, public investment may represent an important contribution to euro area aggregate demand. The countercyclical aspect of this plan is fundamental in order to support solid recovery in the profitability of private business and prompt a strong economic rebound. On the other hand, given its exceptional character, such a recovery plan should shape the long-run development path of the European economy by supporting public investment (e.g. infrastructure) at the European level, as well as country-specific actions in selected strategic areas (e.g. improvements in the healthcare systems or the decarbonisation of European economies). Time is a crucial element. A major investment plan needs to be implemented as soon as the health emergency ends. The later EU institutions and national governments intervene to reignite the private sector's confidence and to counteract the self-reinforcing vicious circle of low demand and production, the harder it will be for the whole European economy to recover.

## Financing the relaunch of the economy

In order to emphasise its EU-wide nature, the recovery plan outlined in the previous section should be implemented and financed by recovery bonds, let us call them European Pandemic Recovery Bonds (EPRBs). EPRBs

2 See Della Posta et al. (2019) for an analysis of investment deficiencies in Europe and the necessity – after the two recessions of 2008-09 and 2012-13 (and even more now, we might add) – of a grand European investment plan that, among other things, could help in restoring a pro-European sentiment (after fiscal austerity and the consequent dreadful social conditions).

differ from public bonds issued in the crisis because they aim at financing the medium- to long-term recovery of the European economy by supporting the technological upgrade and ecological reconversion of its production system. EPRBs could be issued by different European institutions. One option entails the European Investment Bank (EIB) being entitled to issue European recovery bonds. While this option might be the quickest and easiest one to implement as it may imply relatively minor institutional changes, it still requires that the EIB is recapitalised by member states in order to allow it to finance a massive pan-European investment plan. Alternatively, the European Commission could be responsible for the financial support of a more structural and deeper European recovery plan (although this may perhaps be harder for member states to agree on in the short term). This will represent a first significant step towards the creation of a proper fiscal union.

Following Codogno and van den Noord (2020), EPRBs, issued by either the EIB and/or the European Commission, should represent *safe assets* for financial markets eligible for the ECB's asset purchase programmes. The ECB should decide the amount of EPRB-financed expenditures to ultimately cover with money (i.e. by purchasing EPRBs itself), and the amount to leave to investors. In this sense, the ECB should act by taking into account the fine-tuning of eurozone inflationary dynamics with respect to its own inflationary target. Nonetheless, the ECB should also bear in mind that both the ability to meet financial commitments by European firms and governments, i.e. the financial stability of the euro area, and the value of the euro currency ultimately depend on the strength of the underlying production system. All concerns for ECB monetary financing should thus pale in comparison with the urgency to preserve and re-launch the European production system.

Economic concerns may arise from the monetisation of public expenditures. Inflation is not directly linked to the amount of money issued by the central bank, and no major concerns for inflation seem plausible today. Nonetheless, it is important to remember that the present crisis, unlike the Great Recession of 2009, involves some aspects of a potentially relevant supply shock so that, if fought only via demand-side policies (regardless of the financing), undesired levels of inflation may occur. It is precisely for this reason that it is of vital importance to intervene to preserve and relaunch the production potential of the European real economy sector. The much-needed countercyclical fiscal policy should therefore not be limited to relaunching aggregate demand. Still, it is also important to keep in mind that a higher level of inflation than what we have been witnessing in recent years will be desirable to lower the burden of the debt inherited from the crisis. All in all, public spending during the crisis and during the recovery would prevent a further drop in GDP, thereby averting an even greater surge in the public debt-to-GDP ratio. Moreover, a European plan to boost the recovery after the crisis based on investments would play a beneficial role both on the demand side, by increasing aggregate demand that may give rise to an inflationary pressure as time elapses, and the supply side, by enlarging the productive base that instead would contribute to keeping inflation under control.

## An outlook on the future of the eurozone

The project of building a European Union with common markets and institutions has proceeded in alternate phases of great difficulties and great progress in the course of European integration. While fiscal policy has remained anchored to national decisions and inter-governmental coordination due to the overly timid political climate and the obsession with moral hazard, monetary policy under ECB president Draghi greatly changed in order to respond more effectively to the long-lasting consequences of the 2007-08 financial crisis and to the specific problems within the eurozone. An additional step is urgently needed. Fiscal and monetary policy must evolve jointly at the European level. It is not the time for self-imposed restrictions on the spending capacity of the public sector. It is time to abandon dogmas and flawed economic theories on the functioning of monetary systems. With the likely collapse of aggregate demand in the eurozone as a consequence of the fight against the spread of the coronavirus, a new expansion of the ECB's balance sheet to create money is needed, this time to be used in the real economy sector. This monetary expansion will hardly have any significant inflationary impact.[3] And if this were not the case, a (modest) increase in price dynamics might actually make the higher debt burden inherited from this crisis more sustainable.

The temporary suspension of the SGP in the midst of the current health and economic emergency is certainly a positive development. Nonetheless, the ongoing discussions among member states seem to suggest that once the emergency phase is over, pro-austerity countries will push for reintroducing tough fiscal rules and austerity

3 According to Blanchard (2020), a very unlikely sustained increase in inflation in the aftermath of the COVID-19 crisis might be due to a combination of three factors: a very large increase in the debt-to-GDP ratio, larger than the 20-30% or so under current forecasts; a very large increase in the neutral rate, that is the safe real rate needed to keep the economy at potential; perhaps most important, fiscal dominance of monetary policy. Overall, we should be more worried about deflation than inflation.

plans, reiterating the sad story we already saw in the recent past. If we consider that a huge contraction in GDP and a strong increase in government spending jointly contribute to a massive surge in public debt-to-GDP ratio, it becomes clear that the application of SGP – or fiscal compact-inspired fiscal discipline is untenable. We suggest the following alternatives (in ascending order of effectiveness):

1. The crisis-led debt accumulated during the pandemic does not account for the application of fiscal rules once the crisis is over.

2. Bonds issued by national governments during the crisis are fully monetised by the ECB, i.e. they are cancelled or forgiven at maturity.

3. The ECB acts directly as a buyer of last resort, bypassing governments, thus implementing the operations already described above. This helicopter money is calibrated on the need to replace the private economy during the suspension of market activities during the health crisis.

In the long run, European institutions, and eurozone governments in particular, need to be aware that changes are unavoidable, as the current crisis renders fiscal rules and existing treaties outdated and inapplicable. Such long-run structural changes should not be the disorganised results of concessions, but the fruits of a vision for the recovery and development of the economy of the EU as a whole, and of the eurozone in particular. It is time for the eurozone to act as a union. If this is not the case even in the face of such a dramatic crisis and its aftermath, the very existence of the EU will be called into question. In a way, the current COVID-19 emergency may be the last call to make significant steps towards a proper political union.

If even under exceptional conditions disagreements among member states persist regarding the need to act jointly against a huge symmetric shock (with asymmetric impact), then single countries will eventually have to monetise crisis debts by themselves. It goes without saying that this will imply leaving the euro and returning to national central banks, or perhaps moving towards a smaller aggregation of countries agreeing on a deeper sharing of monetary and fiscal policies in a renewed and more cooperative Europe. This might be the case of a Mediterranean European monetary area, perhaps putting together France, Italy and Spain, arising with its own currency, a common fiscal policy and a fully operational central bank. Needless to say, this is a very different type of Europe, and of Economic and Monetary Union, than was originally intended by the noble fathers of European integration.

This is why, in the midst of a tremendous health and economic crisis, we need more than ever full access to all possible joint fiscal and monetary tools. The suspension of the SGP and the instruments proposed in the European Council on April 23rd, as well as the expanding operability of the ECB through PEPP and other measures, give us hope that something is moving in Europe. But, especially for fiscal policy, this is an overly timid step forward. The current discussion among EU member states about the EU recovery fund might represent a promising development towards the creation of a much-needed fiscal space at the EU level supporting an investment-led recovery of the European economy.
